# Identification of Highly Specific scFvs against Total Adiponectin for Diagnostic Purposes

**DOI:** 10.3390/biology6020026

**Published:** 2017-04-26

**Authors:** Peter Wilton, Michael Steidel, Gabriele Krczal, Iris Hermanns, Andreas Pfützner, Alisa Konnerth, Kajohn Boonrod

**Affiliations:** 1 RLP-AgroScience GmbH, AlPlanta-Institute for Plant Research, Breitenweg 71, D-67435 Neustadt, Germany; x96pjw@mun.ca (P.W.); michael.m.steidel@gsk.com (M.S.); gabi.krczal@agroscience.rlp.de (G.K.); Alisa.Konnerth@agroscience.rlp.de (A.K.); 2 University Hospital Mainz, Center of Thrombosis and Homeostasis, D-55131 Mainz, Germany; iris.hermanns@unimedizin-mainz.de; 3 Pfützner Science & Health GmbH, Parcusstr.8, D-55116 Mainz, Germany; andreas.pfuetzner@pfuetzner-mainz.com

**Keywords:** adiponectin, scFv, phage display, diabetes

## Abstract

Adiponectin is one of the most abundant adipokines secreted from adipose tissue. It acts as an endogenous insulin sensitizer and plasma concentrations are inversely correlated with obesity and metabolic syndrome. A decrease in plasma adiponectin levels normally indicates increased hormonal activity of the visceral lipid tissue, which is associated with decreased insulin sensitivity. It may therefore be considered a valuable biomarker for elucidating the underlying deteriorations resulting in type 2 diabetes and macrovascular disease. Here we present the use of phage display technology to identify highly specific antibody fragments (scFvs) against adiponectin. The selected scFvs showed highly specific binding to globular and native adiponectin in ELISA tests. By using our phage display technology, we were able to obtain monoclonal antibodies with specific high affinity binding to the target protein in an effective and easy to upscale manner. The selected scFvs against adiponectin can be used for developing immunoassays suitable for use in metabolic syndrome diagnosis and monitoring.

## 1. Introduction

Amongst the adipokines produced by adipose tissue and expressed as a product of the *ADIPOQ* gene, adiponectin is the most abundant and possibly the most important protein [1,2]. Adiponectin exists in two different forms; high molecular weight (HMW) and low molecular weight (LMW), both of which can be found in blood circulation [3]. HMW adiponectin has been found to have a higher binding affinity to the receptors and to be more physiologically active as compared to the LMW form. It stimulates second messenger activity, which is responsible for mediating the metabolic effects of adiponectin. Suppression of adiponectin is considered a potential biomarker of metabolic syndrome, and the development of type 2 diabetes and macrovascular disease [2,4,5,6,7]. In patients with type 2 diabetes and metabolic syndrome, HMW adiponectin has been found to be a more effective indicator of insulin resistance associated with type 2 diabetes than total plasma adiponectin levels [8]. Snehalatha and co-workers showed that a low adiponectin level in Asian Indians is a strong predictor for development of type 2 diabetes in an otherwise healthy population [9]. The insulin sensitizing properties of adiponectin are considered to be the consequence of AMP-activated protein kinase activation (AMPK), which in turn increases fatty acid (FA) oxidation and hepatic gluconeogenesis [10]. An increase in adiponectin secretion is considered to contribute to the insulin-sensitizing activity of peroxisome proliferator-activated receptor (PPAR)-gamma agonists, such as pioglitazone or rosiglitazone [11,12].

Several monoclonal antibodies for detection of adiponectin are commercially available. Most of them are derived from animals or cell lines. Because of the molecular size and the complexity of the tertiary structure, entire immunoglobulin molecules are very difficult to produce in *Escherichia coli* (*E. coli*) [13]. As a suitable alternative, the scFv protein is one of the smallest functional antibody fragments that maintain antigen-binding activity. It contains only the Fv portion (V_L_ and V_H_) of the antibody molecule [14] and offers more advantages than just being easy to produce in *E. coli*. By employing phage display technology, it is possible to isolate a scFv virtually against any protein. Phage display is the technology that has been used to isolate the majority of recombinant monoclonal antibodies that are currently undergoing clinical evaluation. It is a high-throughput method that is extremely efficient for isolation of highly specific monoclonal antibodies for therapeutic and diagnostic purposes. In this study, we employed phage display against human recombinant adiponectin to select scFvs from a human scFv library (MAC, Cambridge, UK) with very specific and high affinity to globular and native plasma adiponectin.

## 2. Materials and Methods

### 2.1. Expression and Purification of Recombinant Adiponectin

Expression and purification of recombinant adiponectin (Gene Bank accession number ABZ10942.1) in *E. coli* was done according to Richards and coworkers [15]. The gene coding for adiponectin (Seq. GeneID: 9370) from aa 1–246 was synthesized by Geneart (Regensburg, Germany). Subsequently, the full-length gene without signal leader sequence (1–21) was amplified using primers (forward primer 5′-CAGCCATATGGGCCATAATGG-3′ and reverse primer 5′-AACTACATCGA GTAACTCGAGCAC-3′) that introduced *NcoI* and *XhoI* restriction sites. The amplified gene was then inserted to fuse with hexa-histidine (6×His) at the N-terminus into the pET28a+ expression vector at *NcoI* and *XhoI* restriction sites, resulting in pET28a+-His-adiponectin. The plasmid was transformed into *E. coli* BL21 (DE). Cells were grown in LB media containing carbenicillin antibiotics. At OD_600_ = 0.6, protein expression was induced by adding isopropyl-β-d-thiogalactopyranoside (IPTG) up to a final concentration of 1 mM. After incubation at 37 °C for 2 h or at 14 °C overnight, cells were harvested and re-suspended in BugBuster^TM^ Protein Extraction Reagent (Novagen, Merck KGaA, Darmstadt, Germany 10 mL/g of cells) containing 5 µL Benzonase (25 U/µL), 10 mM DTT, and one tablet of complete protease inhibitor (EDTA-free, Roche, Basel, Switzerland). The lysate was centrifuged at 9000× *g* for 10 min, and the soluble and pellet fractions were analyzed by SDS-PAGE for the presence of the expressed protein.

### 2.2. Protein Extraction by Detergent-Based Cell Lysis

Extraction of soluble or inclusion body proteins was performed by using the detergent based protein extraction reagent BugBuster™. The induced cell culture was harvested by centrifugation for 10 min at 12,000× *g* and 4 °C. The cell pellet was re-suspended in BugBuster™ protein extraction reagent (5 mL/g wet cell mass). In addition, lysozyme was added to a final concentration of 0.2 mg/mL, and the mixture was incubated for 20 min at 37 °C. Thereafter, the lysate was sonicated on ice, until viscosity of the sample disappeared. Finally, the protein fractions were centrifuged for 10 min at 12,000× *g* and 4 °C. The soluble protein fraction was isolated by recapturing the supernatant, whereas the inclusion bodies were isolated from the pellet. All fractions were stored at 4 °C.

### 2.3. Purification of Soluble Proteins by Ni-NTA Purification

For purification of soluble proteins, 10 mL of soluble BugBuster™ protein extract was mixed with 1 mL of Protino^®^ Ni-NTA resin (Macherey-Nagel, Düren, Germany). The mixture was stirred slowly on a turn-over shaker for 1 h at 4 °C to let the target fusion proteins bind to the matrix. Thereafter, the resin was filled in a column and the excess fluid was allowed to pass through the filter by gravity. The resin was then washed using 20 bed volumes (~10 mL) of washing buffer (50 mM NaH_2_PO_4_, 300 mM NaCl, 50 mM imidazole, pH 8.0). Subsequently, the resin was mixed with 0.1 mL of elution buffer (50 mM NaH_2_PO_4_, 300 mM NaCl, 250 mM imidazole, pH 8.0), following incubation at room temperature for 30 min. The first eluate was collected and 0.1 mL of elution buffer was added, followed by incubation over night at 4 °C. The eluate was then collected and the elution step was repeated twice to ensure quantitative elution. The purity and quantity of collected eluates were analyzed by SDS-PAGE and western blot analysis. The elution fractions were pooled and the protein concentration was determined by Bradford protein assay, as per the manufacturer’s instructions (Bio-Rad, Hercules, CA, USA). The purified proteins were stored at 4 °C.

### 2.4. Phage Display

ScFv phage libraries (A and B) were obtained from the Medical Research Council (MRC) Centre for Protein Engineering (CPE, Cambridge, UK). The preparations of helper phage, the amplification of the libraries, and the bio-panning were carried out as recommended by the supplier (MRC CPE) with a minor adaptation. In brief, a mix of two phage libraries was panned in 96-well microtiter plates (Nunc Immuno^TM^ Maxisorb^TM^, ThermoFisher Scientific, Waltham, MA, USA). Three wells were coated with recombinant adiponectin (1 µg/well in 1× PBS buffer, 1 M NaCl, 320 mM Na_2_HPO_4_, 170 mM NaH_2_PO_4_, pH 7.2) and left overnight at 4 °C. Three rounds of subsequent bio-pannings were performed. For the first selection round, we used 3 × 100 µL of each library (2 × 10^13^ transformation units (t.u) of phage library A and 3 × 10^13^ t.u of library B). For the second and third round the culture supernatant, which contained the polyclonal phages from the previous round of bio-panning, was used for antigen selection. To ensure enrichment of phage particles the titer of recovered phage of each panning was calculated according to information of the manufacturer. To obtain monoclonal phage scFvs, single colonies from titration plates of the 3rd round were cultured and assayed by ELISA as recommended by the supplier (MRC CPE). Clones that gave the highest signals were selected to infect *E. coli* HB2151 cells to express soluble scFvs. The expressed scFvs from different fractions—culture media and periplasmic space—were analyzed by ELISA and western blot analysis, respectively.

### 2.5. Enzyme-Linked Immuno Sorbent Assay (ELISA)

The presence of scFv was detected by using 100 µL protein-L-conjugated with peroxidase (POD) at a concentration of 1:10,000. The binding signal was induced by the addition of 100 µL peroxidase substrate (3,3′-5,5′-tetramethybenzidine, Sigma-Aldrich, St. Louis, MO, USA) and incubation for 5–20 min (depending on the color intensity). Then the reaction was stopped by adding 50 µL stop solution (2 M H_2_SO_4_) and the absorbance was measured with an ELISA reader at 450 nm.

To determine the expressed monoclonal scFvs in culture media, single clones of the third bio-panning were cultured in microtiter 96-well plates and the scFv expression was induced with IPTG. After centrifugation, 100 µL of the overnight culture media of each expressed clone was coated in a MaxiSorp polystyrene plate (Nunc^TM^, ThermoFisher Scientific) and left at 4 °C overnight. The coated plate was blocked with 2% skim milk in PBS for 2 h. The plate was washed and 100 µL of protein-L POD (1:10,000) were added. After 1 h of incubation, the plate was washed three times with PBS-0.1% Tween and one time with PBS. The binding of scFv-protein-l-POD was determined and measured as described above.

### 2.6. Analysis of Expressed scFvs by SDS-PAGE

The scFvs localized in periplasmic space were extracted using BugBuster^TM^ and 10 µL of total proteins was analyzed by SDS-PAGE via a standard method using 9% polyacrylamide ready-to-use gels (Anamed, Groß-Bieberau, Germany). Gels were either stained with Coomassie blue staining reagent (Fermentas, ThermoFisher Scientific) or were transferred to polyvinylidenfluorid (PVDF) membranes using an electrophoresis transfer system (Bio-Rad). ScFvs were detected using anti-His antibody (Qiagen, Hilden, Germany) followed by anti-mouse conjugated peroxidase (POD). Bound antibodies were detected by enhanced chemiluminescence reaction (Pierce^TM^, ThermoFisher Scientific).

### 2.7. ELISA Detection of Adiponectin—Binding Activity of scFvs

For verification of the binding activity of monoclonal scFvs, meaning their potency to bind adiponectin, the induced cell pellet was extracted with 50 µL/well of BugBuster^TM^ reagent and was incubated for 10 min with shaking. Thereafter, 150 µL/well of 1× PBS was added. After centrifugation, 100 µL of the mixture was used to verify the adiponectin-binding activity in an ELISA test. The binding complex was determined by using protein-l-POD and the signal was developed and measured as described above. To determine the efficiency and specificity of the purified scFv, an ELISA was performed as described above.

### 2.8. Purification of scFvs

*E. coli* HB2105 infected with selected monoclonal phages were grown at 30 °C in 2× TY medium supplement with 1% glucose until OD_600_ = 0.8, followed by induction overnight with 1 mM IPTG. The induced cells were harvested and the soluble scFv, localized in the periplasmic space, was extracted using BugBuster^TM^. The induced bacterial cell pellets from 100 mL of cell culture were re-suspended with 1 mL BugBuster^TM^. The suspension was centrifuged for 10 min at 12,000× *g*. scFvs contained in the periplasmic space were purified with Ni-NTA resin (Macherey-Nagel) as recommended by the manufacturer. The purity of the scFvs was determined by SDS-PAGE followed by a Coomassie Brilliant Blue staining. The concentration of purified scFvs was measured using Bradford reagent (Bio-Rad). The binding activity of purified scFvs to adiponectin was analyzed by ELISA.

### 2.9. Sandwich ELISA Assay

Total adiponectin in plasma samples was detected with Teco’s Total Human Adiponectin ELISA kit (TECO medical AG, Sissach, Switzerland), except that the scFvH5 (0.1 µg/mL) was used as a captor. Plasma of patients obtained from the Institute for Clinical Research and Development (IKFE, Mainz, Germany) was diluted to 1:300 with buffer, as recommended by the manufacturer (TECO medical AG). Recombinant human adiponectin provided in the kit was used as internal calibrator for adiponectin amounts. A standard curve was established by plotting standard concentration on the *x*-axis (linear scale) against the absorbance of the standards on the *y*-axis (linear scale). The adiponectin concentrations in diluted patient sera were calculated from the standard curve.

## 3. Results

### 3.1. Production of Recombinant Adiponectin

**Figure 1:**
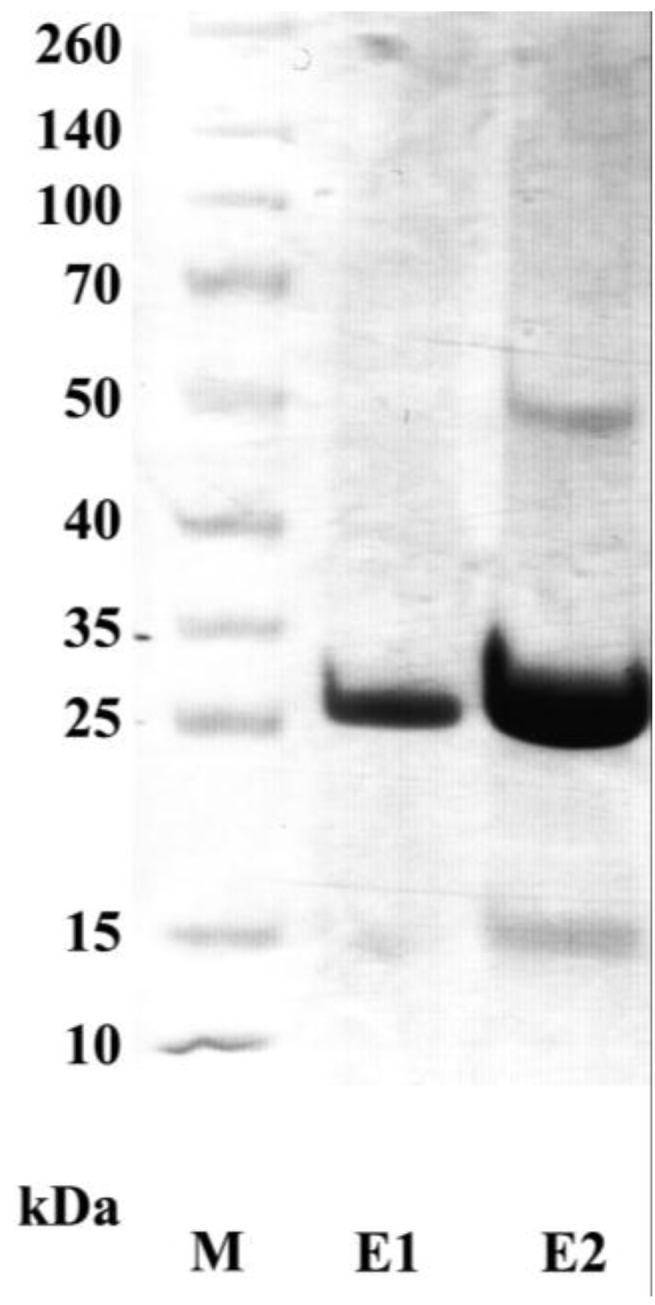
SDS-PAGE analysis of the purified recombinant adiponectin. The full-length adiponectin without LD was expressed as a 6×His fused protein in *E. coli* and was purified using Ni-NTA based purification. After separating in an SDS-PAGE, the gel was stained with Coomassie Brilliant Blue. E1 and E2: eluate 1 and 2, respectively; M: protein marker.

The gene encoding adiponectin was synthesized (Genscript) and the codon usage was optimized for expression in *E. coli*. Attempts to express full-length adiponectin as a soluble protein in *E. coli* failed [16]. As it was shown that expressing adiponectin lacking the 18 amino acids mammalian signal sequence (LD) can be successfully performed in *E. coli* [15], we cloned the adiponectin full-length gene lacking LD into an expression vector (pET 28a+, Novagen) C-terminal fused with a hexa-histidine tag (6×His) for later protein purification. After induction with IPTG, the expressed protein was verified by SDS-PAGE and was stained with Coomassie Brilliant Blue. A clear dominant band of 25 kDa in both soluble and inclusion body fraction was detected, indicating that the recombinant protein was partially expressed as soluble protein (data not shown). The expressed soluble protein was purified using Ni-NTA agarose beads. In 300 µL of eluate, a total of 450 µg of protein was received (1.5 µg/µL, measured by Bradford assay). The purity of the recombinant adiponectin protein was confirmed by SDS-PAGE (Figure 1), and the protein was further used for selecting specific scFvs by bio-panning.

### 3.2. Bio-Panning

Three rounds of bio-panning were performed. In order to obtain a sufficient amount of enriched phage binders from the two supplied libraries, a high amount of recombinant adiponectin (1 µg) was used. The enrichment of adiponectin binders was monitored by calculating the titer of phage recovered after panning. The phage recovery rate (output/input ratio) after the third round was about 200-fold higher than that after the first round, which indicated successful enrichment of adiponectin binders [16]. The monoclonal ELISA was performed after the third round of bio-panning. Individual clones were rescued and examined for antigen-binding activity by a phage ELISA. To get scFv with a high binding activity, we selected monoclonal phages binding adiponectin at 10 times lower concentration (0.1 µg) than used for bio-panning. The results showed that monoclonal phage D1, F1, E6, E1, H5, G5, G6, and G12 (named according to the position of the well in the 96-well plate) exhibited the highest signals in the ELISA [16] and were therefore chosen for production of soluble scFvs.

### 3.3. Production of Soluble scFvs

**Figure 2:**
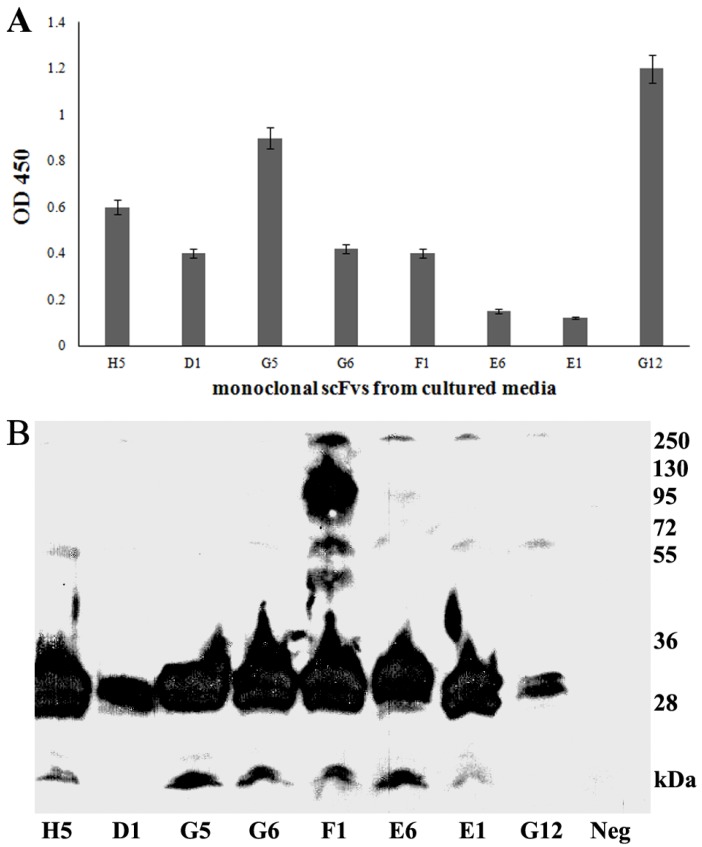
Determination of the expression profiles of the selected unpurified scFvs. The presence and amount of the expressed scFvs was analyzed in an ELISA for the scFvs exported into culture media (100 µL of cell culture supernatant) (**A**) or in a western blot analysis for the scFvs remained in periplasmic space (10 µL of crude protein extract) (**B**).

Plasmids isolated from selected monophage scFv were transformed into HB 2151 *E. coli* cells for soluble scFv expression. Since the scFvs gene was fused to the pelB leader sequence (a signal peptide that leads proteins into periplasmic space and later via secretion out of the cells [17]), all selected clones were induced by IPTG for soluble expression of the scFv antibody. The overnight-induced bacterial culture was harvested by centrifugation, and the supernatant was thereafter determined for protein expression by ELISA. The ELISA results (Figure 2A) suggested that the selected scFvs were expressed in the culture media at low levels, except for scFvs G5 and G12.

### 3.4. Periplasmic Extraction of scFv

**Figure 3:**
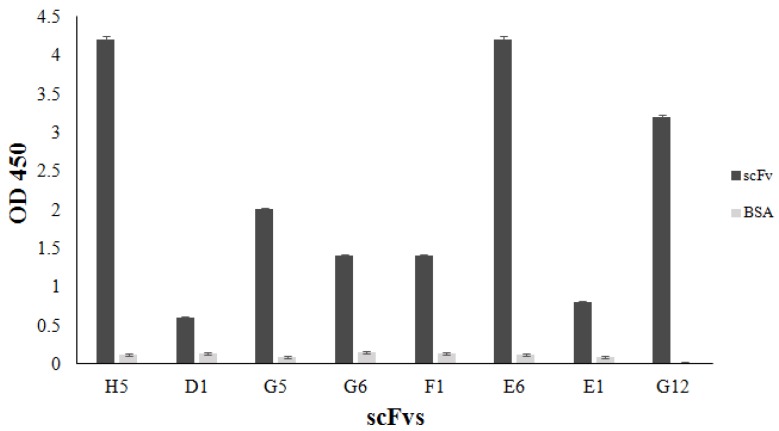
Binding activity for adiponectin of the expressed scFvs. The expressed scFvs were extracted from periplasma and an equal amount of total proteins (10 µg/µL) was used directly for measurement of the adiponectin-binding capacity in an ELISA. ScFvH5 showed the highest binding activity for recombinant adiponectin in ELISA. Bovine serum albumin (BSA) was used as a negative control.

Although scFvs were found to be expressed in low amounts only in the culture media, it could not be excluded that the expressed scFvs either accumulated partially in the periplasmic space or formed inclusion bodies in the cytoplasm. In order to determine the expression pattern of these scFvs, soluble proteins accumulated in periplasmic space of the bacteria were extracted and verified by western blotting (Figure 2B). A clear and strong signal for putative scFv proteins of about 27 kDa was detected in all tested clones, suggesting that most of the expressed scFvs were trapped in the periplasmatic space. Interestingly, signals of 54 and 81 kDa proteins for scFvF1 were also obtained. This may suggest formation of polymeric structures of this expressed scFv. The binding activities of the scFvs, meaning their potency to bind recombinant adiponectin, were further verified by ELISA (Figure 3). All selected scFv clones exhibited different binding to the recombinant adiponectin. scFvH5, exhibiting the highest binding activity, was selected for further analysis.

### 3.5. Purification and Characterization of the scFv Antibody

**Figure 4:**
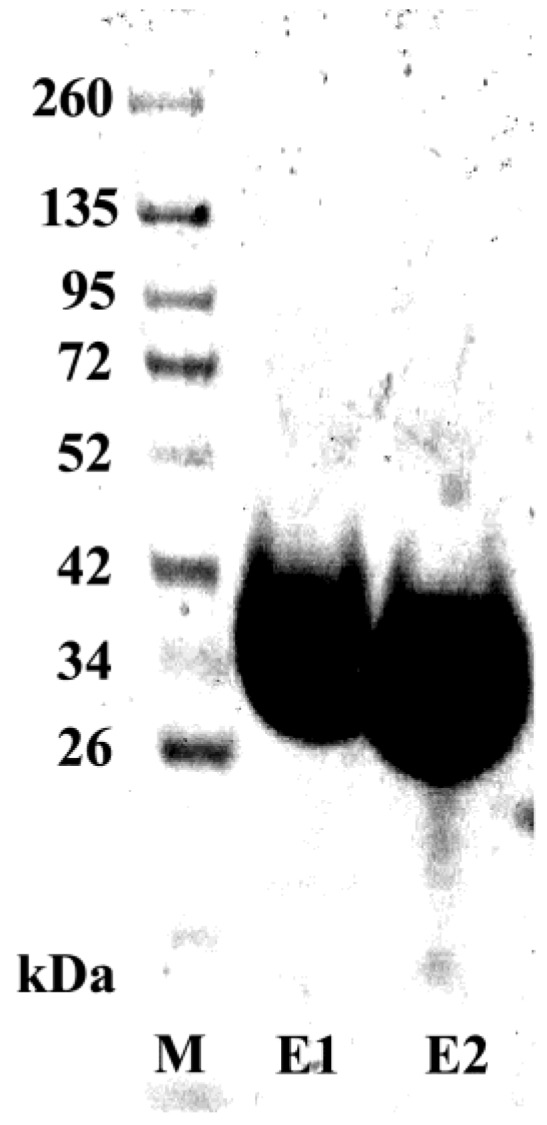
Determination of the purity of purified scFvH5. Purified scFv (2 µg) was subjected to SDS-PAGE followed by Coomassie Brilliant Blue staining. M: protein marker; E1 and E2: first and second eluate.

**Figure 5:**
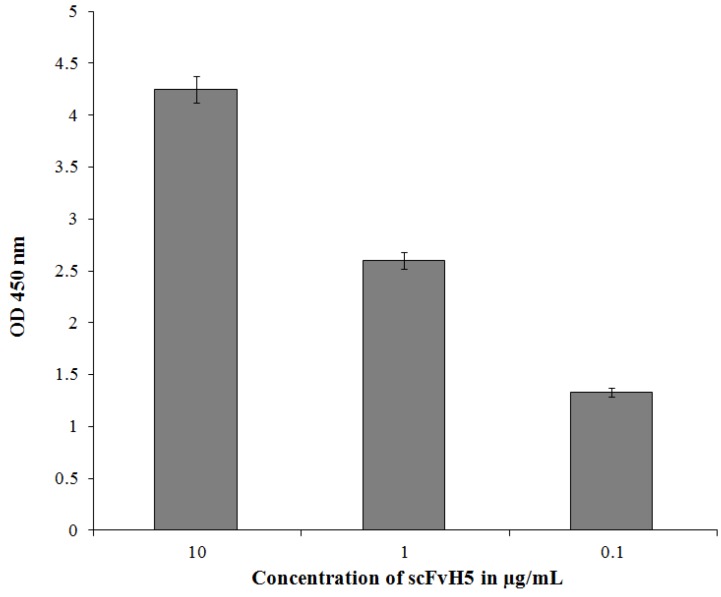
Binding activity of purified scFvH5. Recombinant adiponectin (1 µg) was coated in a microtiter plate. For antigen detection, the purified scFvH5 was serially diluted from 10 µg to 0.1 µg/1 mL. The binding activity of the scFvH5 was detected with protein L-POD. The ELISA results are given as mean ± SD values from triple determinations.

**Figure 6:**
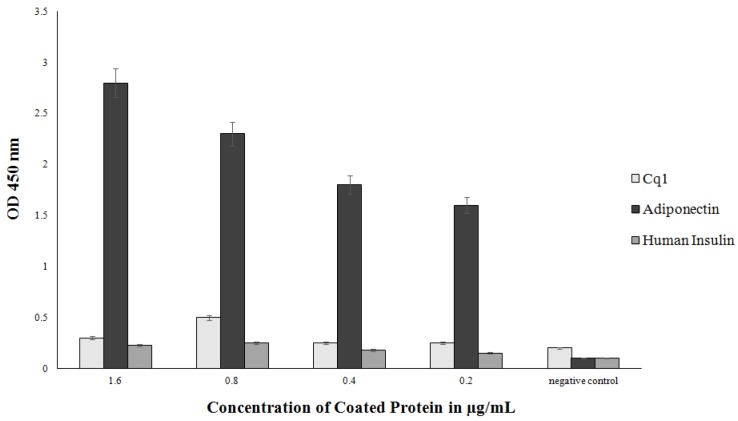
Sensitivity and Specificity of scFvH5. Sensitivity and specificity of scFvH5 were verified by ELISA using 0.1 µg/mL of scFvH5. In addition to the coated recombinant adiponectin, protein Cq1 and human insulin were used for determining the specificity, while the different amounts of the coated proteins were used for determining the sensitivity of scFvH5. The ELISA results are given as mean ± SD values from triple determinations. Insulin was used as a general negative control.

ScFvH5, showing the highest binding activity to recombinant adiponectin, was extracted from the periplasma and purified using Ni-NTA agarose. The purity of the resulting scFv proteins was determined by SDS-PAGE followed by Coomassie brilliant blue staining (Figure 4). The binding activity of scFvH5 was assessed by ELISA using a serial dilution (1, 1:10, and 1:100) of 10 µg/mL of the purified scFv antibody. The results indicated that scFvH5 binds to the recombinant adiponectin in a dose-dependent manner (Figure 5). A scFvH5 concentration of 0.1 µg/1 mL can detect the recombinant adiponectin even at the lowest concentration used (0.2 µg/mL), indicating a high binding activity (Figure 6). Adiponectin is structurally homologous to complement 1q (C1q), a subcomponent of complement C1, which is highly abundant in human serum [18]. Therefore, the scFvH5 was further tested for cross-reactivity with a recombinant C1q in an ELISA. The result indicated that scFvH5 had no cross-reactivity with C1q (Figure 6).

### 3.6. Binding of the Selected scFvH5 to Native and Globular Adiponectin

**Figure 7:**
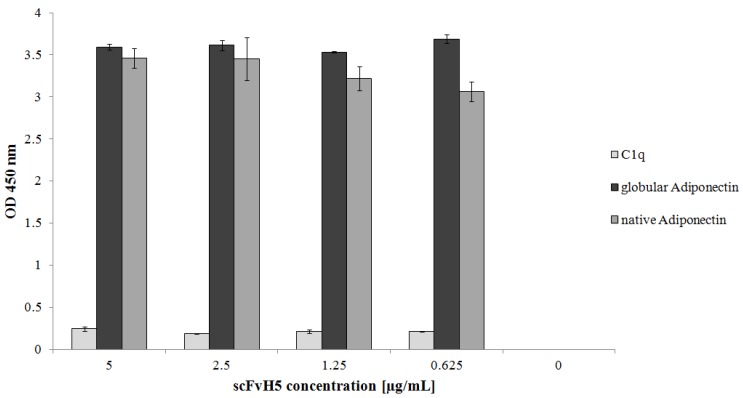
The purified scFvH5 can bind to globular and native adiponectin. In an ELISA, 100 µL of recombinant globular or native adiponectin (Biovendor) or C1q as negative control (1 µg/mL each) were coated on a microtiter plate and different concentrations of scFvH5 were used for antigen. The ELISA results are given as mean ± SD values from triple determinations.

The tertiary structure of native adiponectin could potentially deviate from that of our human recombinant protein. Therefore, we investigated the binding activity of selected scFvs to native adiponectin isolated from human serum (Biovendor) and to the recombinant globular adiponectin (Biovendor) by ELISA. The result indicated that scFvH5 could detect all tested adiponectin antigens (Figure 7).

### 3.7. Binding of scFvH5 to Adiponectin in Human Plasma

A complex, heterogeneous protein mixture such as proteins in plasma or serum would be less suitable for coating a plate for direct ELISA detection unless the protein of interest is over-expressed and thus the major protein present in the sample. As this is not the case for adiponectin, the combination of scFvH5 as a capture antibody and a commercial antibody (TECO) as a detector in a sandwich ELISA assay were used to confirm that the scFvH5 can detect adiponectin in human plasma. The result showed that scFvH5 could indeed detect total adiponectin in human plasma (Table 1).

The amount of total adiponectin was extrapolated from a calibration curve using the recombinant human adiponectin provided by the kit (TECO). The ELISA in the “reference method” was done by using mAb provided by the kit as a captor. The measurements were performed in duplicates.

## 4. Discussion

Adiponectin secretion in humans is suppressed by enhanced hormonal activity of the visceral lipid tissue, which is induced by weight gain and which is associated with the development of metabolic syndrome and type 2 diabetes mellitus [2]. For diagnostic purposes, several monoclonal anti-adiponectin antibodies are commercially available, which have been developed based on animal and cell line expression. This production method, however, is costly and time consuming. In this study, we employed phage display technology and scFv libraries to generate and characterize specific scFvs against adiponectin. We performed three bio-panning selection procedures on immobilized recombinant adiponectin. To save the high cost for commercially available adiponectin we expressed a partially soluble recombinant adiponectin (with truncated LD) in *E. coli*, as previously reported by Richards and co-workers [15], which could be purified through a hexa-histidine fused tag. While some unspecific proteins were found in the eluates, the protein was found to be pure enough for phage display. It was shown that human adiponectin is highly glycosylated, and three oligomeric forms were differentially glycosylated [19,20]. Posttranslational modification of adiponectin consists of hydroxylation and glycosylation of several conserved lysine residues in the collagenous domain of adiponectin. Therefore, using the recombinant adiponectin (non-glycosylated) as an antigen for phage display will avoid the selection of scFvs that would specifically detect the different glycosylation patterns of the different forms of adiponectin. On the contrary, our approach will result in scFvs targeting non-glycosylated domains of adiponectin and thus are able to detect different forms of adiponectin. After three rounds of bio-panning, we selected monoclonal phages that bound adiponectin in a 10-time lower concentration than the concentrations used in bio-panning. This step reduced unspecific and weak binding phages. At the end, seven monoclonal phages showing the highest binding activity in the ELISA were obtained for soluble protein production. Most of the resulting scFvs were trapped in the periplasmic space, while one of them (scFvG12) was highly excreted in the media. Expression of scFv in the periplasmic space, however, carries numerous advantages. First and foremost, expression in the periplasmic space allows proper folding to occur. The periplasm, unlike the bacterial medium, is an oxidizing environment, in contrast to the reducing environment of the cytoplasm where assembly and disulfide bond formation can occur. [21]. Second, for up-scaling production, it is much more effective to purify proteins extracted from the periplasm without a concentration step, as required when purifying from culture media. The finally purified scFvH5 was very efficient for detecting adiponectin even at very low concentrations in ELISA (20 ng). Moreover, it exhibited very specific binding to all tested adiponectin (recombinant, globular, and native) and no cross-reactivity with the structurally most alike protein Cq1. As scFvH5 binds to all forms of tested adiponectin, it is tempting to speculate that the binding epitope of scFvH5 is in the globular structure. The result of the sandwich ELISA proved that scFvH5 can bind adiponectin in human plasma. Moreover, the result indicates that scFvH5 can recognize not only the immobilized protein, but also free soluble protein.

The advantages of using scFv instead of complete antibodies, particularly for therapeutic or diagnostic uses, are addressed in various publications [22]. One important advantage is the small size, enabling efficient production of a ready-to-use protein after genetic engineering and by employing bacterial expression processes. In the present study, scFvs with specific binding to recombinant, globular, and native adiponectins were generated by phage display. To our knowledge, this is the first report to discuss using this technique for effective generation of scFvs against adiponectin. Anti-adiponectin scFv can now be used in immunological studies, including fluorochrome conjugation [23], bispecific antibody production, bifunctional protein synthesis, and other techniques of genetic engineering. Further studies are needed to evaluate the potential suitability of recombinant anti-adiponectin scFv for diagnostic applications.

## 5. Conclusions

The role of adiponectin in influencing insulin sensitivity and body fat contents is a matter of extensive research in the field of complex metabolic diseases such as obesity and type 2 diabetes. Physiological levels of adiponectin regulate insulin sensitivity by increasing FA-metabolism and energy expenditure in muscle and have anti-inflammatory effects, while decreased adiponectin levels have been shown to correlate with development of insulin resistance.

For diagnostic purposes, several monoclonal anti-adiponectin antibodies, which have been developed based on animal and cell line expression, are already commercially available. We here show the first study employing phage display selection and bacterial expression for generating an antibody fragment (scFv) against adiponectin. Our finally purified scFvH5 exhibited very specific binding to adiponectin (recombinant, globular, and native) and no cross-reactivity with the structurally most alike protein Cq1. In preliminary tests, it also proved its potential to detected human adiponectin in patient samples (plasma) with a high sensitivity. Further studies are needed to evaluate the potential suitability of recombinant anti-adiponectin scFv for diagnostic applications.

## Figures and Tables

**Table 1:** Sandwich ELISA assay of total adiponectin in human plasma using scFvH5 as a captor.

Plasmas		scFv H5 Sandwich-ELISA (µg/mL)	Reference Method TECO- Assay (µg/mL)
Patient sample 1	Value 1	15.23	16.95
	Value 2	15.09	15.80
Patient sample 2	Value 1	2.06	1.67
	Value 2	2.06	1.67
Patient sample 3	Value 1	2.80	3.19
	Value 2	2.80	3.19
Patient sample 4	Value 1	7.11.	4.08
	Value 2	6.81	3.82
